# Supplementary material for: Diagnostic and Prognostic Value of microRNAs in Patients with Laryngeal Cancer: A Systematic Review
Source: Noncoding RNA. 2023 Jan 19;9(1):9. doi: 10.3390/ncrna9010009 (PMC9966707; doi:10.3390/ncrna9010009)
Supplement: Supplementary file 1 [file ncrna-09-00009-s001.zip › ncrna-2149719-supplementary.pdf]

Table S1: Tissue miRNAs associated with diagnostic features in LSCC.

Tissue miRNA	Dysplasia	LSCC	Grading	T stage	N Stage	Number of Paper(s)*	Cohort numerosity (range)#	Refs
**Downregulated**								
let-7a-5p	0	3↓	2↓	1↓	1↓	3	150 (43-59)	[110, 122, 181]
let-7c-5p	0	1↓	0	0	0	1	57	[34]
let-7f-5p	0	1↓	0	0	0	1	32	[146]
miR-101-3p	0	1↓	0	1↓	1↓	1	80	[156]
miR-107	0	2↓	1↓	0	1↓	2	70 (30-40)	[22, 72]
miR-10a-5p	0	1↓	0	0	0	1	32	[146]
miR-1205	0	1↓	0	1↓	1↓	1	44	[70]
miR-1225-5p	0	1↓	0	0	0	1	20	[96]
miR-124-3p	0	1↓	0	1↓	0	1	34	[85]
miR-125a-5p	0	1↓	0	0	0	1	30	[180]
miR-125b-2	0	1↓	0	0	0	1	10	[140]
miR-125b-5p	0	6↓	1↓	2↓	1↓	6	292(12-87)	[32, 43, 82, 101, 117, 130]
miR-1287-5p	0	1↓	0	0	0	1	49	[137]
miR-128a-3p	0	1↓	0	0	0	1	10	[140]
miR-1299	0	1↓	0	0	0	1	10	[140]
miR-133a-3p	0	2↓	0	0	0	2	138 (62-76)	[28, 184]
miR-133b	0	3↓	0	0	0	3	210 (50-84)	[28, 46, 131]
miR-134-5p	0	0	0	1↓	0	1	42	[41]
miR-136-5p	0	2↓	0	0	0	2	50 (10-40)	[64, 140]
miR-139-5p	0	2↓	0	1↓	0	2	156 (76-80)	[28, 134]
miR-1-3p	0	1↓	0	0	0	1	76	[28]
miR-140-3p	0	1↓	0	0	0	1	76	[28]
miR-140-5p	0	3↓	0	0	0	3	146 (40-56)	[68, 83, 192]
miR-141-3p	0	1↓	1↓	1↓	1↓	1	60	[73]
miR-143-3p	0	2↓	2↓	2↓	1↓	2	112 (52-60)	[90, 93]
miR-144-3p	0	3↓	0	0	0	3	116 (24-60)	[42, 146, 173]
miR-145-5p	0	9↓	2↓	3↓	2↓	9	662 (12-188)	[32, 42, 59, 92, 99, 130, 131, 153, 167]
miR-147a	0	1↓	0	0	0	1	45	[24]
miR-148a-5p	0	1↓	0	0	0	1	12	[32]
miR-149-5p	0	2↓	1↓	1↓	1↓	2	176 (33-143)	[43, 179]

miR-152-3p	0	1↓	0	1↓	1↓	1	83	[144]
miR-153-3p	0	1↓	0	0	0	1	38	[170]
miR-154-5p	0	1↓	1↓	1↓	0	1	104	[100]
miR-16-5p	0	1↓	0	0	0	1	10	[129]
miR-1911-3p	0	1↓	0	0	0	1	78	[29]
miR-193a-3p	0	1↓	0	0	0	1	10	[129]
miR-194-5p	0	1↓	0	0	0	1	20	[81]
miR-195-5p	0	7↓	1↓	4↓	2↓	7	580 (23-182)	[75, 107, 111, 114, 115, 146, 197]
miR-199b-5p	0	1↓	0	0	0	1	87	[82]
miR-203a-3p	0	2↓	0	1↓	1↓	2	92 (32-60)	[146, 175]
miR-204-5p	0	5↓	0	2↓	1↓	5	243 (12-87)	[32, 49, 65, 82, 113]
miR-206	0	4↓	1↓	1↓	1↓	4	128 (10-68)	[126, 140, 158, 193]
miR-210-5p	0	1↓	0	0	0	1	45	[27]
miR-218-5p	0	2↓	0	0	0	2	53 (10-43)	[118, 140]
miR-22-3p	0	2↓	0	1↓	0	2	10	[76] [129]
miR-299-5p	0	1↓	0	0	0	1	10	[140]
miR-29c-3p	0	2↓	1↓	1↓	1↓	2	153 (66-87)	[74, 82]
miR-302b-3p	0	2↓	0	0	0	2	171 (85-86)	[52, 86]
miR-30a-5p	0	1↓	0	0	0	1	10	[140]
miR-330-3p	0	2↓	0	0	0	2	87 (32-55)	[56, 58]
miR-337-3p	0	1↓	0	0	0	1	10	[140]
miR-340-3p	0	1↓	0	0	0	1	30	[38]
miR-340-5p	0	2↓	0	0	0	2	65 (15-50)	[54, 162]
miR-34b-3p	0	1↓	0	0	0	1	10	[140]
miR-34c-3p	0	1↓	0	0	0	1	10	[140]
miR-363-3p	0	1↓	0	0	0	1	48	[98]
miR-365a-3p	0	1↓	0	0	0	1	58	[44]
miR-370-3p	0	2↓	0	0	0	2	58 (18-40)	[139, 195]
miR-372-3p	0	1↓	0	0	1↓	1	76	[28]
miR-375-5p	0	1↓	0	0	0	1	46	[147]
miR-381-3p	0	1↓	0	0	0	1	52	[71]
miR-384	0	1↓	0	0	0	1	12	[89]
miR-410-3p	0	1↓	0	0	0	1	10	[140]
miR-432-5p	0	1↓	0	0	0	1	10	[140]
miR-4497	0	1↓	0	0	0	1	30	[102]

miR-449a	0	2↓	0	0	1↓	2	20 (10-10)	[28, 129]
miR-449b-5p	0	1↓	0	0	0	1	76	[28]
miR-4640-5p	0	1↓	0	0	0	1	30	[94]
miR-4687-3p	0	1↓	0	0	0	1	60	[42]
miR-4726-5p	0	1↓	0	0	0	1	30	[94]
miR-4735-3p	0	1↓	0	0	0	1	36	[51]
miR-4768-3p	0	0	0	1↓	0	1	48	[121]
miR-486-3p	0	2↓	0	0	0	2	40 (10-30)	[37, 140]
miR-486-5p	0	1↓	0	0	0	1	20	[97]
miR-493-3p	0	1↓	0	0	0	1	34	[69]
miR-497-5p	0	1↓	0	0	0	1	38	[30]
miR-506-3p	0	1↓	0	1↓	1↓	1	62	[87]
miR-518a-3p	0	1↓	0	0	0	1	60	[47]
miR-519a-3p	0	1↓	1↓	1↓	1↓	1	96	[135]
miR-524-5p	0	1↓	0	1↓	0	1	20	[48]
miR-539-5p	0	1↓	0	0	0	1	76	[28]
miR-613	0	1↓	0	0	0	1	30	[109]
miR-625-5p	0	1↓	0	1↓	1↓	1	86	[88]
miR-627-5p	0	1↓	0	0	1↓	1	76	[28]
miR-652-3p	0	1↓	0	0	1↓	1	76	[28]
miR-654-3p	0	1↓	0	0	0	1	44	[55]
miR-655-3p	0	1↓	0	0	0	1	105	[23]
miR-663a	0	1↓	0	0	0	1	10	[129]
miR-6786-5p	0	1↓	0	0	0	1	10	[129]
miR-744-5p	0	1↓	0	0	0	1	65	[194]
miR-7-5p	0	1↓	0	0	0	1	30	[95]
miR-766-5p	0	1↓	0	1↓	0	1	60	[40]
miR-873-5p	0	1↓	0	0	0	1	28	[35]
miR-885-5p	0	3↓	0	0	0	3	133 (10-76)	[28, 140, 168]
miR-936	0	1↓	1↓	1↓	1↓	1	25	[62]
**Upregulated**								
let-7b-5p	0	1↑	0	0	0	1	10	[129]
miR-1246	0	2↑	0	0	0	2	73 (12-61)	[32, 61]
miR-1260b	0	0	1↑	0	0	1	60	[42]

miR-129-5p	0	1↑	0	1↑	1↑	1	36	[138]
miR-1297	0	1↑	0	0	0	1	10	[129]
miR-130b-3p	0	1↑	0	0	0	1	60	[42]
miR-130b-5p	0	1↑	0	0	0	1	84	[131]
miR-132-3p	0	2↑	0	0	0	2	86 (10-76)	[28, 183]
miR-142-3p	0	2↑	0	0	0	2	122 (46-76)	[28, 159]
miR-146a-5p	0	1↑	0	0	0	1	33	[196]
miR-147b-3p	0	1↑	0	0	0	1	76	[28]
miR-155-5p	1↑	7↑	4↑	4↑	2↑	7	1246 (20-840)	[26, 28, 59, 84, 106, 128, 169]
miR-17-5p	0	1↑	0	1↑	0	1	39	[66]
miR-181b 5p	0	2↑	0	0	0	2	72 (12-60)	[32, 42]
miR-182-5p	0	1↑	0	0	0	1	76	[28]
miR-183-5p	1↑	1↑	0	0	0	1	20	[26]
miR-184	0	1↑	0	0	0	1	76	[28]
miR-185-5p	0	1↑	0	0	0	1	76	[28]
miR-196a-5p	0	3↑	0	0	0	3	207 (41-84)	[59, 131, 177]
miR-196b-3p	0	1↑	1↑	1↑	1↑	1	79	[91]
miR-196b-5p	0	3↑	1↑	1↑	0	3	230 (41-113)	[28, 103, 177]
miR-198	0	1↑	0	0	0	1	76	[28]
miR-19a-3p	0	3↑	2↑	2↑	1↑	3	166 (10-83)	[140, 142, 165]
miR-202-3p	0	0	0	1↑	0	1	48	[121]
miR-20a-5p	0	1↑	0	0	0	1	10	[129]
miR-20b-5p	0	2↑	0	0	0	2	181 (76-105)	[28, 31]
miR-210-3p	0	4↑	0	0	0	4	174 (12-82)	[26, 32, 42, 59]
miR-21-3p	0	5↑	1↑	0	0	6	252 (12-82)	[32, 42, 59, 97, 146, 168]
miR-214-3p	0	1↑	0	0	0	1	10	[129]
miR-21-5p	1↑	14↑	3↑	3↑	3↑	14	680 (10-116)	[26, 28, 59, 67, 110, 123, 129, 130, 140, 145, 147, 159, 174, 176]
miR-218-3p	0	1↑	0	0	0	1	20	[26]
miR-221-3p	0	2↑	0	1↑	2↑	2	132 (50-82)	[59, 120]
miR-222-3p	0	1↑	0	0	0	1	82	[59]
miR-223-3p	0	1↑	0	0	0	1	46	[159]
miR-23a-3p	0	2↑	0	1↑	1↑	2	134 (52-82)	[155, 160]
miR-23b-3p	0	1↑	0	0	0	1	10	[129]

miR-27a-3p	0	3↑	1↑	1↑	1↑	3	122 (10-62)	[129, 187, 191]
miR-28-5p	0	1↑	0	0	0	1	76	[28]
miR-301a-3p	0	2↑	0	1↑	0	2	196 (76-120)	[28, 163]
miR-31-3p	0	1↑	2↑	0	1↑	2	142 (60-82)	[42, 59]
miR-324-3p	0	1↑	0	0	0	1	76	[28]
miR-331-5p	0	1↑	0	0	0	1	76	[28]
miR-34b-5p	0	1↑	0	0	0	1	33	[196]
miR-34c-5p	0	1↑	0	0	0	1	33	[196]
miR-424-5p	0	1↑	0	0	1↑	1	82	[59]
miR-425-5p	0	2↑	0	0	0	2	76	[28, 80]
miR-449a-5p	0	1↑	0	0	0	1	33	[196]
miR-455-3p	0	1↑	0	0	0	1	84	[131]
miR-455-5p	0	2↑	0	0	0	2	104 (20-84)	[97, 131]
miR-490-3p	0	1↑	0	0	0	1	76	[28]
miR-503-5p	0	2↑	0	0	0	2	60 (12-48)	[32, 112]
miR-512-3p	0	1↑	0	0	1↑	1	76	[28]
miR-525-5p	0	1↑	0	0	0	1	47	[168]
miR-545-3p	0	1↑	0	0	1↑	1	76	[28]
miR-548aa	0	0	0	1↑	0	1	48	[121]
miR-552-3p	0	1↑	0	1↑	0	1	29	[60]
miR-590-5p	0	1↑	0	0	0	1	76	[28]
miR-632	0	1↑	0	0	0	1	10	[105]
miR-6510-3p	0	1↑	0	0	0	1	33	[50]
miR-657	0	1↑	0	0	0	1	49	[137]
miR-708-5p	0	1↑	0	0	0	1	48	[130]
miR-744-3p	0	1↑	0	0	1↑	1	41	[177]
miR-886-3p	0	1↑	0	0	0	1	76	[28]
miR-892a	0	1↑	0	0	0	1	17	[25]
miR-92a-3p	0	1↑	0	0	0	1	87	[82]
miR-93-5p	0	5↑	1↑	1↑	1↑	5	199 (12-60)	[32, 42, 97, 130, 148]
miR-9-5p	0	2↑	1↑	1↑	1↑	2	135 (32-103)	[150, 186]
miR-96-5p	0	3↑	0	0	1↑		129 (20-76)	[28, 50, 97]
miR-98-5p	0	1↑	0	0	0	1	10	[77]
**Other**								

miR-106b-3p	1↑	5↑ 1↓	0	1↑	2↑	6	145↑ (14-59) 48↓	[26, 127, 132, 145, 146, 196]
miR-1290	0	1↑ 1↓	0	0	1↓	2	50↑ 48↓	[161, 196]
miR-138-5p	0	1↑ 1↓	0	1↓	0	2	76↑ 30↓	[28, 166]
miR-139-3p	0	1↑ 2↓	0	0	1↓	3	48↑ 72↓(25-47)	[36, 168, 196]
miR-148a-3p	0	1↑ 1↓	0	0	1↓	2	179↑ 12↓	[182, 178]
miR-181a-5p	0	1↑ 2↓	0	0	0	3	76↑ 45↓	[28, 45, 78]
miR-18a-5p	0	1↑ 1↓	0	0	0	2	12↑ 10↓	[32, 129]
miR-205-5p	0	3↑ 2↓		1↓	0	5	98↑ (10-48) 40↓ (10-30)	[129, 130, 136, 164, 188]
miR-24-3p	0	1↑ 2↓	0	0	0	3	10↑ 35↓ (15-20)	[125, 129, 152]
miR-26a-5p	0	1↑ 2↓	0	0	0	3	56↑ 38↓ (10-28)	[53, 108, 129]
miR-29a-3p	0	1↓	0	1↑	0	2	48↑ 28↓	[119, 121]
miR-300	0	1↑ 1↓	0	0	0	2	133↑ 30↓	[185, 190]
miR-31-5p	0	2↑ 1↓	1↑	1↑ 1↓	0	3	142↑ (60-82) 40↓	[42, 59, 104]
miR-34a-5p	0	1↑ 3↓	2↓	2↓	2↓	4	33↑ 150↓ (10-71)	[50, 124, 129, 172]
miR-375-3p	0	2↑ 7↓	3↓	1↑ 6↓	1↑ 1↓	9	227↑ (48-179) 316↓ (10-76)	[28, 140, 145, 149, 159, 178, 188, 196]

*Numbers correspond to the number of studies reporting the association with the diagnostic parameter. ↑ : upregulated miRNA. ↓: downregulated miRNA. #Cohort numerosity indicates the number of patients, and when two or more papers reported the same miRNA, the minimum and the maximum number of patients is indicated inside brackets.
